# The Family as Part of the Client’s System in the Wards Psychiatric Hospitals : A Comparative Study Evaluating the Implementation of a Family-Centered Care Model

**DOI:** 10.1192/j.eurpsy.2023.242

**Published:** 2023-07-19

**Authors:** R. Shor, A. Shalev

**Affiliations:** 1 The School of Social Work and Social Welfare, The Hebrew University of Jerusalem, Jerusalem; 2 Faculty of Social Work, Ashkelon Academic College, Ashkelon, Israel

## Abstract

**Introduction:**

Family caregivers can be a valuable source of knowledge and help in treating persons with mental illness during a psychiatric hospitalization and in ensuring a continuity of service between family caregivers and professionals after the discharge form a psychiatric hospital. Therefore, a family care model has been developed in order to provide professional staff members in psychiatric wards guidelines for how to collaborate with family caregivers during each stage of the hospitalization of their family member with mental illness.

**Objectives:**

To examine the impact of implementing the Family Care model in psychiatric hospitals in Israel on the family caregivers and on the continuity of care between the hospitalization and the community.

**Methods:**

A comparative study was conducted implementing an AB design with an intervention and control groups. Seventy five persons participated in the control group and 93 in the intervention group. Questionnaires were delivered to family caregivers during the hospitalization and after the discharge from hospitalization, about the quality of collaboration of the family caregivers with the professionals during the hospitalization, about the family caregivers health and mental health, about their knowledge and ability to handle situations related to the family member with SMI and about the continuity of services between the hospitalization and the community

**Results:**

The findings indicate that in the intervention group comparing to the control group there was: A lower level of anxiety of the family caregivers after the discharge of their family member from the psychiatric hospital, a higher level of evaluation of the caregivers’ knowledge how to respond to the needs of the family members with mental illness, a higher level of evaluation of the quality of collaboration between the caregivers and the professional staff during the psychiatric hospitalization and a more frequent contact a between the persons with mental illness and the mental health services as well as a greater compliance with treatment after the discharge from the psychiatric hospital.

**Conclusions:**

The Family-Centered care model expands the traditional boundaries of the definition of the patient in psychiatric hospitals to include the family caregivers. This model could help prevent the development of problems for the family caregivers and it could help improve the continuation of services in the community. Therefore, the findings support the implementation of this model in psychiatric hospitals.

**Disclosure of Interest:**

None Declared

